# Guideline-discordant inhaler regimens after COPD hospitalization: associations with rurality, drive time to care, and fragmented care – a United States cohort study

**DOI:** 10.1016/j.lana.2023.100597

**Published:** 2023-09-21

**Authors:** Arianne K. Baldomero, Ken M. Kunisaki, Chris H. Wendt, Carrie Henning-Smith, Hildi J. Hagedorn, Ann Bangerter, R. Adams Dudley

**Affiliations:** a Pulmonary, Allergy, Critical Care, and Sleep Medicine, Minneapolis VA Health Care System, Minneapolis, MN, USA; b Pulmonary, Allergy, Critical Care, and Sleep Medicine, University of Minnesota, Minneapolis, MN, USA; c Center for Care Delivery and Outcomes Research, Minneapolis VA Health Care System, Minneapolis, MN, USA; d Division of Health Policy and Management, University of Minnesota, Minneapolis, MN, USA

**Keywords:** Drive time to care, Access to care, Health care disparity, Veterans affairs, Rural health

## Abstract

**Background:**

Many patients receive guideline-discordant inhaler regimens after chronic obstructive pulmonary disease (COPD) hospitalization. Geography and fragmented care across multiple providers likely influence prescription of guideline-discordant inhaler regimens, but these have not been comprehensively studied. We assessed patient-level differences in guideline-discordant inhaler regimens by rurality, drive time to pulmonary specialty care, and fragmented care.

**Methods:**

Retrospective cohort analysis using national Veterans Health Administration (VA) data among patients who received primary care and prescriptions from the VA. Patients hospitalized for COPD exacerbation between 2017 and 2020 were assessed for guideline-discordant inhaler regimens in the subsequent 3 months. Guideline-discordant inhaler regimens were defined as short-acting inhaler/s only, inhaled corticosteroid (ICS) monotherapy, long-acting beta-agonist (LABA) monotherapy, ICS + LABA, long-acting muscarinic antagonist (LAMA) monotherapy, or LAMA + ICS. Rural residence and drive time to the closest pulmonary specialty care were obtained from geocoded addresses. Fragmented care was defined as hospitalization outside the VA. We used multivariable logistic regression models to assess associations between rurality, drive time, fragmentated care, and guideline-discordant inhaler regimens. Models were adjusted for age, sex, race/ethnicity, Charlson Comorbidity Index, Area Deprivation Index, and region.

**Findings:**

Of 33,785 patients, 16,398 (48.6%) received guideline-discordant inhaler regimens 3 months after hospitalization. Rural residents had higher odds of guideline-discordant inhalers regimens compared to their urban counterparts (adjusted odds ratio [aOR] 1.18 [95% CI: 1.12–1.23]). The odds of receiving guideline-discordant inhaler regimens increased with longer drive time to pulmonary specialty care (aOR 1.38 [95% CI: 1.30–1.46] for drive time >90 min compared to <30 min). Fragmented care was also associated with higher odds of guideline-discordant inhaler regimens (aOR 1.56 [95% CI: 1.48–1.63]).

**Interpretation:**

Rurality, long drive time to care, and fragmented care were associated with greater prescription of guideline-discordant inhaler regimens after COPD hospitalization. These findings highlight the need to understand challenges in delivering evidence-based care.

**Funding:**

10.13039/100000002NIH10.13039/100006108NCATS grants KL2TR002492 and UL1TR002494.

**Research in context**

**Evidence before this study**

We searched PubMed and Google Scholar for studies reporting on guideline-based inhaler regimens in chronic obstructive pulmonary disease (COPD). We used the following search terms (“inhalers”) AND (“guideline” OR “guideline adherence” OR “practice guidelines”) AND (“chronic obstructive pulmonary disease OR pulmonary disease, chronic obstructive). Our search was limited to studies among adults which were published between January 1, 2000 and July 30, 2023. Our search identified 39 studies. Many studies were clinical practice guidelines and studies quantifying and evaluating trends in prescription of inhaler therapies in COPD. At least three studies assessed factors associated with prescription of guideline-recommended inhaler regimens in COPD. In observational studies, prescription of guideline-discordant inhaler regimens is common among patients discharged after hospitalization for COPD exacerbations—patients at highest risk for poor subsequent health outcomes. Living in a rural area and fragmented care are increasingly recognized as potential factors that contribute to poor health outcomes in COPD, however, few studies have evaluated how these factors are associated with receipt of guideline-concordant COPD management. Additionally, to our knowledge, no prior studies have investigated how travel requirements to receive health services, such as pulmonary specialty care, are associated with receipt of guideline-discordant COPD inhaler regimens.

**Added value of this study**

To address gaps in the literature, we used the national Veterans Health Administration (VA) electronic health record data to examine receipt of guideline-discordant inhaler regimens among patients discharged from the hospital for COPD exacerbation by rurality, drive time to the closest pulmonary specialty care, and fragmented care. This work could help tailor health system efforts by contributing to our understanding of how factors such as geographical access to and fragmentation of health care are associated with delivery of guideline-discordant care for patients with COPD.

**Implications of all the available evidence**

Prescription of guideline-discordant inhaler regimens was common after hospitalization for COPD hospitalization and was associated with living in a rural area, longer drive time to pulmonary specialty care, and fragmented care. Our findings suggest that access to health care and challenges in care coordination are potentially contributing to suboptimal delivery of evidence-based COPD care, highlighting the need to develop effective methods to health care delivery to target COPD patients with these risk factors.

## Introduction

Inhaled medications for chronic obstructive pulmonary disease (COPD) reduce the risk of hospitalizations and improve quality of life.^1^, ^2^, ^3^ However, evidence-based clinical practice guideline recommendations for inhaled therapies in COPD frequently are often not followed in clinical practice.^4^, ^5^, ^6^, ^7^, ^8^ Guideline-concordant inhaler regimens, in addition to tobacco cessation, immunizations, and pulmonary rehabilitation, are particularly important for patients following hospitalization for COPD exacerbations. The COPD patients at highest risk for poor subsequent outcomes, including increased mortality and accelerated lung function decline, are those recently hospitalized for a COPD exacerbation.^9^^,^^10^ Nonetheless, prescription of guideline-discordant inhaler regimens to these patients is common.^11^, ^12^, ^13^, ^14^

Geographic factors—such as living in a rural area—and fragmented care are increasingly recognized as potential factors that contribute to poor health outcomes in COPD.^15^, ^16^, ^17^, ^18^ These factors likely impact health care access, but few studies have evaluated whether these factors are associated with prescription of guideline-concordant COPD inhaler regimens.^12^^,^^19^

This study aimed to evaluate differences in prescription of guideline-discordant inhaler regimens after hospitalization for COPD exacerbation by rurality, drive time to the closest pulmonary specialty care, and whether care was fragmented using the United States Veterans Health Administration (VA) national data. We hypothesized that guideline-discordant inhaler regimens would be more common in patients: 1) living in a rural area, 2) with longer drive times, and 2) with fragmented care.

## Methods

### Data sources

We obtained patient data from the VA Corporate Data Warehouse (CDW). The VA CDW is a network of national databases that incorporates data from multiple data sets throughout the Veterans Health Administration into one standard database structure to facilitate reporting and data analysis at the enterprise level.^20^ We used VA Inpatient files to extract VA hospitalization data. To capture hospitalizations paid for by the VA but received outside the VA (VA-purchased care), we used the Non-VA Care Program Integrity Tools and Fee Basis files. Data for inhalers prescribed by a VA provider were acquired from VA Pharmacy files. This cohort study followed the Strengthening the Reporting of Observational Studies in Epidemiology (STROBE) reporting guidelines.

### Study population

**Fig. 1:**
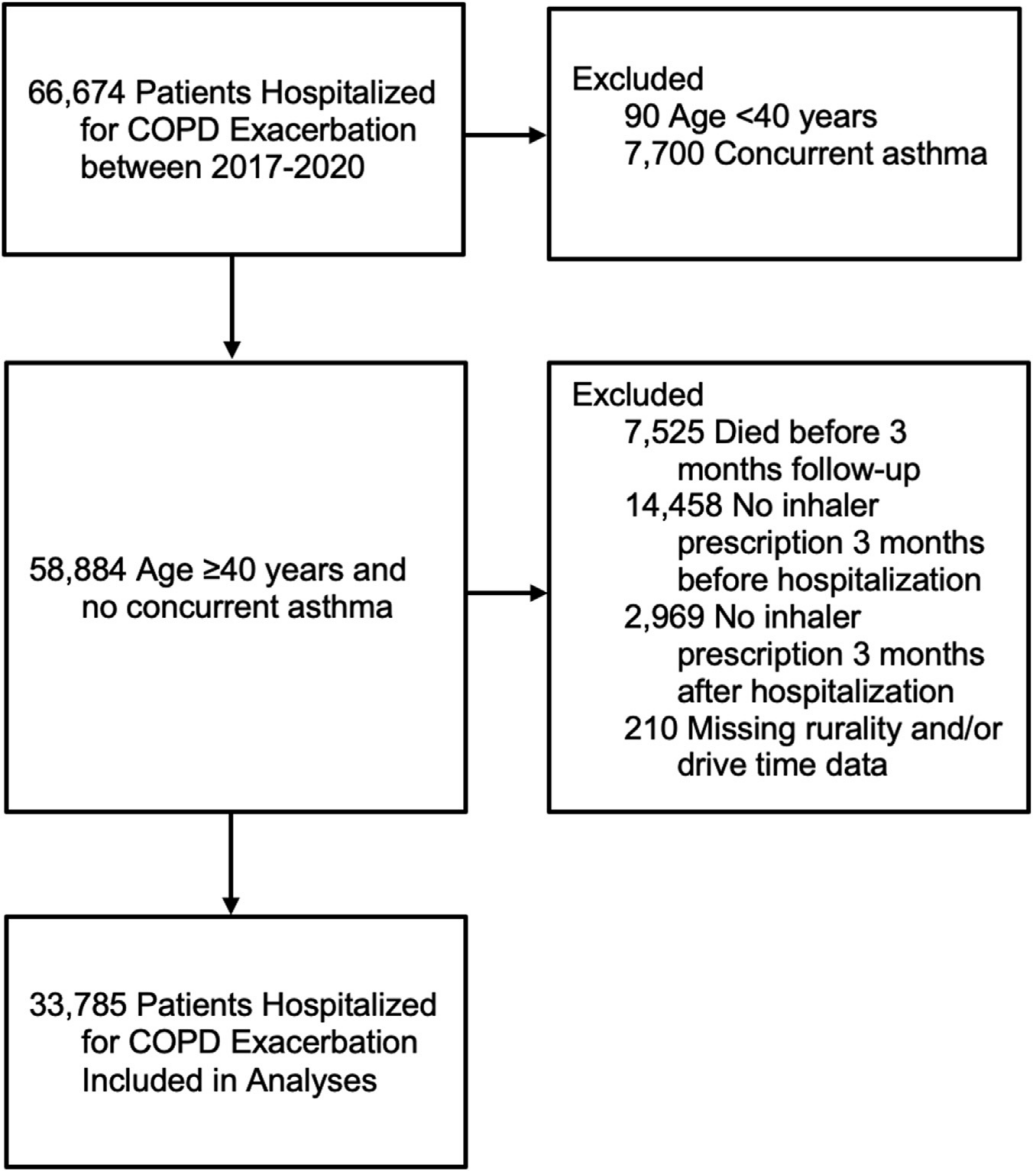
**Flow diagram of patients hospitalized for COPD exacerbation included in the analyses**.

We identified patients hospitalized for acute COPD exacerbation between January 2016 and December 2019 (Fig. 1). We included patients aged ≥40 years with at least one discharge with *International Classification of Diseases-Tenth Revision* (ICD-10) codes for: 1) COPD as the primary diagnosis (J40, J41, J41.0, J41.1, J41.8, J42, J43, J43.0, J43.1, J43.2, J43.8, J43.9, J44, J44.0, J44.1, or J44.9), or 2) respiratory failure as primary (J96, J96.0, J96.00–J96.02, J96.2, J96.20–J96.22, J96.9, J96.90–J96.92, or R06.03) and COPD exacerbation as a secondary diagnosis (J44.0 or J44.1).^21^ The first hospitalization within the study period was the index hospitalization. We excluded patients who had a concurrent diagnosis of asthma and those who died prior to the follow-up (within 3 months after discharge). Some VA patients may also receive prescriptions from non-VA providers; therefore, patients who were not receiving inhaler prescriptions from the VA prior to the index hospitalization were excluded (Supplementary Table S5).

### Guideline-discordant COPD inhaler regimens

We applied guideline recommendations for COPD inhaler regimens from the 2014 and 2021 Veterans Health Administration (VA) and Department of Defense (DoD) CPG^22^^,^^23^; the 2020 ATS CPG^24^; and the 2017, 2018, 2019, 2020 Global Initiative for Chronic Obstructive Lung Disease (Supplementary Table S1).^25^, ^26^, ^27^, ^28^ Long-acting inhalers are recommended as maintenance therapy. Specifically, combination long-acting muscarinic antagonist and long-acting beta-agonist (LAMA + LABA) or combination LAMA, LABA, and inhaled corticosteroid (LAMA + LABA + ICS) is recommended for patients with an exacerbation in the past year; therefore, we defined these regimens as guideline-concordant. Conversely, guideline-discordant inhaler regimens were defined as any other regimen (short-acting inhaler/s only, ICS monotherapy, ICS + LABA, LABA monotherapy, LAMA monotherapy, or LAMA + ICS).

### Outcomes

The primary outcome was prescription of guideline-discordant inhaler regimens at 3 months after hospitalization. We selected 3 months as the time period to allow sufficient time for outpatient clinic follow-up and inhaler optimization after hospital discharge. To assess if inhaler therapy patterns change over time, we also assessed guideline-discordant inhaler regimens at 6 months after hospital discharge as a secondary outcome.

### Exposure variables

#### Rurality

Geocoded patient residential addresses, urban and rural designations, and drive times from the patients residential address to VA facilities were collected from the VA Planning Systems Support Group Geocoded Enrollee Files.^29^ Each patient's residential address is designated by the VA as urban or rural using United States Rural-Urban Commuting Area (RUCA) codes^30^ with ‘urban’ as RUCA codes 1.0 or 1.1 and ‘rural’ as all others. This definition of ‘urban’ is narrow, incorporating only census tracts with a metropolitan area core, but is consistent with definitions used in VA policy and other research.^29^^,^^31^

#### Drive time to the closest VA pulmonary specialty care

Drive time estimates from each patient's address to the closest VA facility where pulmonary specialty care was available were calculated using geospatial technologies as described by the VA Health Economics Resource Center and were based on expected driving routes, traffic, and average driving conditions.^29^^,^^32^

#### Fragmented care

We defined fragmented care as hospitalization outside the VA, but paid for by the VA (VA-purchased care) among patients who receive primary care and prescriptions from the VA.^18^

#### Covariates

We used the Area Deprivation Index (ADI), which includes census block-level income, education, employment, and housing quality metrics, as a measure of socioeconomic status.^33^ We categorized ADI into ≤20th percentile, 21st–40th, 41st–60th, 61st–80th, 81st–100th for analysis. We used the Charlson Comorbidity Index (CCI) to assess health status.^34^ We included geographic region—Midwest, Northeast, South, and West— as a covariate to account for regional variation in practice.^35^^,^^36^

In the U.S. Veteran Health Administration, the majority of patients who are hospitalized for any indication typically receive 1-month supply of medications on discharge. After that first month post-discharge, outpatient VA providers, most commonly primary care VA providers, are responsible for continuing, renewing, or changing prescriptions. This means that, in the case of COPD, the inhalers prescribed at discharge can differ from the ones prescribed starting one month after discharge. In addition, based on our prior work, we found that many patients living in rural areas have their COPD hospitalizations in non-VA hospitals, which we are referring to in this paper as fragmented care.^36^ We use this term because being hospitalized outside the VA system can frequently result in lack of information regarding hospital course, including newly-prescribed medications reaching VA primary care providers.

### Statistical analysis

We evaluated the characteristics of patients who were prescribed guideline-discordant inhaler regimens after hospitalization for COPD exacerbation. We calculated the percentage who received guideline-discordant inhaler regimens (a binary outcome at the patient level) and used the exact (Clopper-Pearson) confidence limits to estimate the 95% confidence interval. We used multivariable logistic regression models to estimate the individual associations between prescription of guideline-discordant inhaler regimens and: 1) rurality; 2) drive time (categorized into ≤30 [referent group], 31–60, 61–90 and > 90 min); and 3) fragmented care. We ran the logistic analyses for each exposure variable as separate models to aid in identification of geographic risk factors and patient populations who could be targeted for future interventions. We also wanted to assess if these exposure variables could be predictive on their own. We additionally performed sensitivity analyses including all three exposure variables in one model (Supplementary Table S6). Multivariable logistic regression models were adjusted for age, sex, race/ethnicity, CCI, ADI, and health care facility region to minimize the potential confounding effects of sociodemographic factors, underlying comorbidities, and regional variability in clinical practices (Supplementary Tables S7–S11). We used an omnibus likelihood-ratio chi-square test to assess whether a predictor was associated with prescription of guideline inhaler regimens. We additionally assessed multicollinearity among the three exposure variables by examining the variable correlation matrix and variance inflation factors in a multivariable regression model (Supplementary Tables S11–S13). We did not observe high correlation (e.g. coefficients of ≥0.8) among the three exposure variables and the variance inflation factors were not large (values between 1.02 and 1.30), suggesting few issues stemming from multicollinearity. We also performed secondary analyses evaluating the relationships between sex, race/ethnicity, and inhaler regimens (Supplementary Table S14).^37^, ^38^, ^39^ Finally, we assessed interactions between exposure and explanatory variables which showed significant interactions between rurality and race, rurality and region, and fragmented care and race; there was no significant interaction between drive time and exposure variables (Supplementary Table S15). We then performed stratified analyses for the groupings with significant interactions (Supplementary Tables S16 and S17). All statistical analyses were performed using SAS software, version 9.4 (SAS Institute).

### Study oversight

This study was approved and the requirement for informed consent was waived by institutional review boards at the Minneapolis VA Health Care System (VAM-20-00583) and the University of Minnesota (STUDY00011069).

### Role of the funding source

The funders of the study had no role in study design, data collection, data analysis, data interpretation, or writing of the report.

## Results

The baseline characteristics of the patients are in Table 1. Among 33,785 patients who received their primary care and prescriptions from the VA and had a hospitalization for COPD exacerbation, the mean age was 70.5 ± 8.3 years, 26,893 (79.6%) White, 4660 (13.5%) Black/African American, and 13,606 (40.3%) had CCI of 3 or higher. More than 14,360 (42.5%) were assigned to primary care facilities in the Southern region, 8663 (25.6%) Midwest, 6474 (19.2%) West, and 4219 (12.5%) Northeast regions. More than 12,705 (36.7%) lived in a rural area and 21,692 (64.2%) had to drive more than 30 min to the closest VA pulmonary specialty care. Twenty-nine percent had fragmented care (Table 1).

**Table 1:** Baseline characteristics of patients hospitalized for COPD exacerbation.

	Guideline-discordant n = 16,398	Guideline-concordant n = 17,387	Total N = 33,785
**Age** (years), mean ± SD	70.7 ± 8.5	70.3 ± 7.7	70.5 ± 8.1
**Male,** No. (%)	15,741 (96.0)	16,744 (96.3)	32,485 (96.2)
**Race/Ethnicity,** No. (%)			
American Indian/Alaska Native	145 (0.9)	162 (0.9)	307 (0.9)
Asian	29 (0.2)	36 (0.2)	65 (0.2)
Black/African American	2101 (12.8)	2559 (14.7)	4660 (13.5)
Native Hawaiian/Pacific Islander	111 (0.7)	101 (0.6)	212 (0.7)
White	13,190 (80.4)	13,703 (78.8)	26,893 (79.6)
Unknown/Declined	822 (5.0)	826 (4.8)	1648 (4.9)
**Charlson Comorbidity Index**^a^**,** No. (%)			
0	1108 (6.8)	1138 (6.6)	2246 (6.7)
1–2	8296 (50.6)	9637 (55.4)	17,933 (53.1)
≥3	6994 (42.7)	6612 (38.0)	13,606 (40.3)
**Comorbidities**^a^***,*** No. (%)			
Congestive Heart Failure	3413 (20.8)	2978 (17.1)	6391 (18.9)
Cardiovascular Accident	888 (5.4)	920 (5.3)	1808 (5.4)
Dementia	4,28 (2.6)	336 (1.9)	764 (2.3)
Diabetes	5659 (34.5)	4986 (28.7)	10,645 (31.5)
Liver Disease	1271 (7.8)	1286 (7.4)	2557 (7.6)
Malignancy	2287 (14.0)	2523 (14.5)	4810 (14.2)
Myocardial Infarction	869 (5.3)	805 (4.6)	1674 (5.0)
Peripheral Vascular Disease	1569 (9.0)	1458 (8.9)	3027 (9.0)
Renal Disease	2069 (12.6)	1647 (9.5)	3716 (11.0)
**Tobacco Use Status,** No. (%)			
Current Smoker	7081 (43.2)	7469 (43.0)	14,550 (43.1)
Former Smoker	4330 (26.4)	4988 (28.7)	9318 (27.6)
Never Smoker	1391 (8.5)	1236 (7.1)	2627 (7.8)
Unknown	3596 (21.9)	3694 (21.3)	7290 (21.6)
**Region**^b^, No. (%)			
Midwest	4269 (26.0)	4394 (25.3)	8663 (25.6)
Northeast	1846 (11.3)	2373 (13.7)	4219 (12.5)
South	7267 (44.3)	7098 (40.8)	14,365 (42.5)
West	2985 (18.2)	3489 (20.1)	6474 (19.2)
**Area Deprivation Index**^c^ (percentile), mean ± SD	63.4 ± 23.7	60.7 ± 24.9	62.0 ± 24.3
**Rurality**, No. (%)			
Urban	9792 (59.7)	11,288 (64.9)	21,080 (62.4)
Rural	6606 (40.3)	6099 (35.1)	12,705 (36.7)
**Drive Time to the Closest Pulmonary Specialty Care,** No. (%)			
≤30 min	5370 (32.8)	6723 (38.7)	12,093 (35.8)
31–60 min	3594 (21.9)	3974 (22.9)	7568 (22.4)
61–90 min	2232 (13.6)	2218 (12.8)	4450 (13.2)
>90 min	5202 (31.7)	4472 (25.7)	9674 (28.6)
**Fragmented Care**^d^**,** No. (%)			
No	10,903 (66.5)	13,113 (75.4)	24,016 (71.1)
Yes	5495 (33.5)	4274 (24.6)	9769 (28.9)

a Charlson Comorbidity Index (CCI) scores range from 0 to 33, with higher scores indicating greater disease burden and increased risk of death within 1 year.^40^ List of comorbidities were based from CCI.

b Geographic regions were divided into four categories according to each patient's Veterans Integrated Services Networks (VISN) which are regional systems of care working together to meet local health care needs and provides access to care. Midwest includes patients from VISNs 10, 15, 12, and 23; Northeast from VISNs 1, 2, and 4; South from VISNs 5, 6, 7, 8, 9, 16, and 17; and West from VISNs 19, 20, 21, and 22.^35^ Sixty-four patients (0.2%) had missing VISN data.

c Area Deprivation Index provides percentile ranking of neighborhoods by census block groups based on the aggregated domains of income, education, employment, and housing quality (percentile ranged from 1 to 100, with higher scores indicating higher levels of socioeconomic disadvantage).^33^

d Fragmented care was defined as hospitalization in a non-VA health care facility, but paid for by the VA (VA-purchased care), among patients who receive primary care and prescriptions at the VA.^18^

**Fig. 2:**
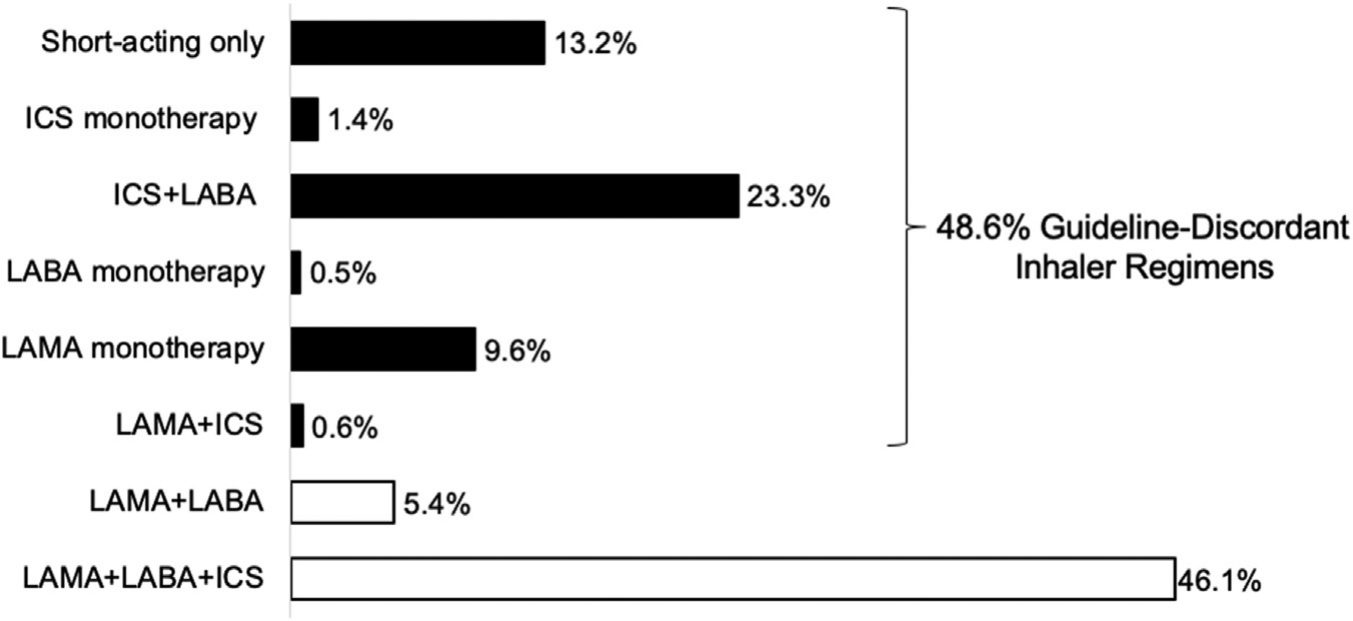
**Inhaler regimens at 3 months after COPD hospitalization (N = 33,785)**. *Abbreviations:* ICS, inhaled corticosteroid; LABA, long-acting beta-agonist; and LAMA, long-acting muscarinic antagonist. Black bars represent guideline-discordant inhaler regimens. White bars represent guideline-concordant inhaler regimens.

Guideline-discordant inhaler regimens were prescribed to 16,389 or 48.6% (95% Confidence Interval [CI]: 48.0%–49.1%) of patients at 3 months after discharge (Fig. 2). Rural residents had higher adjusted odds of guideline-discordant inhaler regimens than urban residents (adjusted odds ratio [aOR] 1.15 [95% CI: 1.12–1.23]) (Table 2). Similarly, longer drive time to the closest pulmonary specialty care was associated with increasing odds of receiving discordant inhaler regimens. Compared to patients with drive times ≤30 min, patients with drive time 31–60 min had aOR 1.09 (95% CI: 1.03–1.16), 61–90 min had aOR 1.19 (95% CI: 1.10–1.27), and >90 min had aOR 1.38 (95% CI: 1.30–1.46). Fragmented care was associated with higher odds of guideline-discordant inhaler regimens (aOR 1.56 [95% CI: 1.48–1.63]) (Table 2).

**Table 2:** Logistic regression analyses for guideline-discordant inhaler regimens at 3 Months after COPD hospitalization (N = 33,785).

	Guideline-discordant inhaler regimens^a^^,^^b^ n = 16,398	
	Adjusted rate, %	Adjusted odds ratio
	(95% Confidence Interval)	
**Rurality**		
Urban (ref)	48.2 (45.3–51.2)	1.00
Rural	52.3 (49.2–55.4)	1.18 (1.12–1.23)
**Drive Time to the Closest Pulmonary Specialty Care**		
≤30 min (ref)	46.6 (43.5–49.6)	1.00
31–60 min	48.8 (45.7–51.9)	1.09 (1.03–1.16)
61–90 min	50.8 (47.5–54.1)	1.19 (1.11–1.27)
>90 min	54.5 (51.4–57.6)	1.38 (1.30–1.46)
**Fragmented care**^c^		
No (ref)	45.8 (42.8–48.8)	1.00
Yes	56.8 (53.7–59.8)	1.56 (1.48–1.63)

a Models were adjusted or age, sex, race/ethnicity, Charlson Comorbidity Index (CCI), Area Deprivation Index (ADI), and region.

b Guideline-discordant nhaler regimens include: short-acting inhalers only, ICS monotherapy, ICS + LABA, LABA monotherapy, LAMA monotherapy, or LAMA + ICS.

c Fragmented care was dfined as hospitalization in a non-VA health care facility, but paid for by the VA (VA-purchased care), among patients who receive primary care and prescriptions at the VA.^18^

The most commonly prescribed inhaler regimen was the guideline-concordant combination LAMA + LABA + ICS (15,574; 46.1%), followed by two guideline-discordant regimens ICS + LABA (7884; 23.3%) and short-acting inhalers only (4451; 13.2%) (Fig. 2, Supplementary Table S2, and Supplementary Table S3). In sensitivity analyses evaluating individual inhaler regimens, living in a rural area, longer drive time to the closest pulmonary specialty care, and fragmented care were similarly associated with higher odds of prescription of short-acting inhalers only and ICS + LABA; however, we did not see significant associations with LAMA monotherapy (Supplementary Table S3).

At 6 months after COPD hospitalization, the percentage of patients with guideline-discordant inhaler regimens decreased to 12,188 or 38.3% (95% CI: 37.7%–38.8%, Supplementary Figure S1). Living in a rural area, longer drive time to pulmonary specialty care, and fragmented care remained associated with higher odds of guideline-discordant inhaler regimens at 6 months (Supplementary Table S4).

Female patients had higher odds of guideline-discordant inhaler regimens after COPD hospitalization compared to male patients (aOR 1.14 [95 CI: 1.02–1.28]) (Supplementary Table S14). Compared to patients hospitalized for a COPD exacerbation who were White, Black/African American patients had lower odds of guideline-discordant inhaler regimens (aOR 0.81 [95% CI: 0.76–0.86]) (Supplementary Table S14). We did not find significant differences in prescription of guideline-discordant inhaler regimens among other races/ethnicities compared to White patients, which could partially be explained by the very small percentages of patients from some other race/ethnic groups in this predominantly White VA cohort (<1% American Indian/Alaska Native, Native Hawaiian/Pacific Islander, and Asian). Compared to patients living in urban areas, Black/African American and White patients living in rural areas had higher odds of guideline-discordant inhaler regimens (aOR 1.46 [95% CI: 1.22–1.73] and aOR 1.16 [95% CI: 1.10–1.22], respectively) (Supplementary Table S16). Additionally, Black/African American and White patients who had fragmented care had higher odds of guideline-discordant inhaler regimens compared to those who did not have fragmented care (aOR 1.88 [95% CI: 1.62–2.18) and aOR 1.52 [95% CI: 1.44–1.60]) (Supplementary Table S17). Compared to patients in urban regions, patients in rural Midwest, Northwest, and West regions had higher odds of guideline-discordant inhaler regimens (Supplementary Table S16).

## Discussion

In this national cohort of COPD patients, prescription of guideline-discordant inhaler regimens was common after hospitalization for COPD exacerbation and was associated with living in rural areas, longer drive time to pulmonary specialty care, and fragmented care. Our findings suggest that access to health care and challenges in care coordination are potentially contributing to suboptimal delivery of evidence-based COPD care in this high-risk patient population. Our data highlight the need to develop effective methods of health care delivery to target COPD patients with these risk factors.

Other investigators have reported a wide range of rates of guideline-discordant inhaler regimen prescriptions. Yip et al. reported that 54% of patients discharged after COPD hospitalization in the New-York Presbyterian Healthcare System did not receive long-acting inhalers (defined as no ICS, ICS + LABA, or LAMA).^41^ Gershon et al. and Poon et al. found that only 12% received guideline-discordant inhaler regimens (defined as not receiving LAMA or ICS + LABA) among patients hospitalized for COPD exacerbation or patients with ≥2 COPD exacerbations in Ontario, Canada.^12^^,^^42^ Among COPD patients discharged from hospitals in Italy, Di Martino et al. reported that 35% received guideline-discordant inhaler prescriptions, defined as not receiving LAMA or LABA.^13^ These mixed results are likely due to differences in inhaler regimens considered as guideline discordant. Because these studies included patients from different years, the variation in regimens considered as guideline discordant in these studies likely reflect temporal evolution of guideline recommendations. In our study, we defined guideline-discordant inhaler regimens as not receiving LAMA + LABA or LAMA + LABA + ICS based on application of clinical practice guidelines and recommendations from multiple organizations (Supplementary Table S1). Factors that have been associated with guideline-discordant inhaler regimens include older age and higher comorbidity burden; however, geographic factors and fragmented care have not been extensively evaluated.^12^^,^^13^

We found that living in a rural area and/or having a longer drive time to pulmonary specialty care were associated with prescription of guideline-discordant inhaler prescriptions. These findings are consistent with our previous work showing that underutilization of spirometry was associated with longer drive time to a site offering this service, which was more common among patients living in rural areas.^19^ These studies suggest that geographic barriers to accessing health care services are possible mechanisms by which rural-urban disparities might arise. Importantly, we also show the separate associations between rurality and drive time to accessing care; while they are related, urban residents can also have long drive times, which may impact the quality of their care. Our findings show the need to focus on these two distinct, albeit related, geographic factors.

We also found that fragmented care (hospitalization in a non-VA health care facility, but paid for by the VA, among patients who receive primary care and prescriptions at the VA) was associated with guideline-discordant inhaler therapy regimens. We did not find prior studies that examined the association of fragmented care with concordance of COPD prescriptions with guideline recommendations. However, Rinne et al. found in a national VA cohort of hospitalized COPD patients that the odds of readmission after hospitalization for COPD was higher among Veterans who were hospitalized outside the VA (aOR 1.20 [95% CI: 1.03–1.40]).^18^ Our findings that fragmented care was associated with higher risk of guideline-discordant prescriptions offers a potential causal mechanism for Rinne et al.‘s observation.

Our findings are not isolated to COPD. For example, rural patients experience worse health outcomes compared to urban patients in a wide range of conditions.^43^, ^44^, ^45^ Additionally, rurality and longer travel time and/or distance to care were associated with lower rates of guideline-concordant diagnostic testing, screening/preventive care and therapies in multiple conditions, including cancer, osteoporosis, heart disease, kidney disease, and obstetric care.^19^^,^^46^, ^47^, ^48^, ^49^ These suggest geographic barriers to care are broadly predictive of receiving lower quality of care and having worse outcomes.

The VA is the largest integrated health care system in the U.S., and many VA patients receive all their care through the VA. This includes not just primary care, specialty care, and hospital services, but also low or no cost prescriptions. To mitigate health care access issues related to rurality and drive time to care, the VA already uses multiple strategies such as virtual or telehealth options, addition of satellite clinics in rural areas transportation services to VA health care facilities, travel reimbursement, and lodging options on VA property proximal to health care facility.^19^ Other potential solutions could be considered. For example, health systems could identify patients living in a rural area, those with longer drive time to care, and recent hospitalization in non-VA facilities and offer additional support when setting up appointments, including specialty care visit. Leveraging the skills of other allied health care providers, such as pharmacists, respiratory therapists, and nursing in providing guideline-adherent care can also be explored. Another consideration is investment in broadband infrastructure to expand telehealth services. We need to test novel, targeted solutions in health care delivery to improve guideline-concordant care for these high-risk populations. Future research is needed to evaluate potential interventions prior to widespread implementation.

However, the VA also pays for veterans in emergency situations (such as an acute COPD exacerbation) to go to a non-VA hospital. This provides more access, options, and flexibility, but can also lead to challenges in sharing information, coordinating care, and maintaining continuity of care.^36^^,^^50^ To alleviate issues related to fragmented care, potential solutions that could be considered include improving efforts to provide services for patients to access care within the VA system creating incentives for patients, providers, and health care systems to reduce fragmented care, and increasing support to coordinate care between VA and non-VA systems.

Historical data have shown that access to and receipt of health care services (e.g. cancer screening, reproductive health, etc.) are substantially lower among racial/ethnic minorities compared to White individuals.^51^, ^52^, ^53^, ^54^ This pattern has been observed specifically in COPD care provided outside the VA. A national U.S. survey showed that Black/African American, American Indian, and Hispanic respondents with COPD were less likely to receive preventive care such as immunizations than White respondents.^55^ Spirometry testing to confirm a diagnosis of COPD was also significantly more common for White than Hispanic than White (83.6% vs. 59.4%, p = 0.006). In contrast, in our current study assessing care for patients covered by the VA system, we found that minorities did not receive lower quality of care than White patients. In fact, prescription of guideline-discordant inhaler regimens after hospitalization for COPD exacerbation was lower for Black/African Americans vs. White patients, indicating that Black/African patients had received better care. This is consistent with data from Mularski et al., who found that Black/African Americans received higher quality COPD care after adjusting for other covariates including insurance.^56^ Two other studies among veterans with COPD did not show significant differences in receipt of COPD-related services by race/ethnicity, but no studies have shown that patients from minority groups receive worse quality of care than White patients.^19^^,^^57^ A potential explanation for the difference in findings between studies done within the VA and those done outside the VA population is insurance coverage. All the patients in our study receive health care coverage from the VA, whereas health care coverage in non-VA settings may vary significantly across race/ethnicity, with higher proportion of uninsured or underinsured rates among minority groups.

The literature about sex differences in inhaler prescriptions in COPD includes conflicting results. Some studies report that females are more likely to receive maintenance therapies, likely related to more respiratory symptoms among females, than males.^38^ Other studies report that males are more likely to receive guideline-concordant inhaler therapies.^39^ In our study, females who were hospitalized for COPD exacerbation were more likely to be prescribed guideline-discordant inhaler regimens. Reasons for this are unclear, though potential explanations could include delay in or under diagnosis of COPD in females and differences in prescribing habits of providers.^40^

Our study has several important limitations. First, the inhaler data we used relied on VA pharmacy data. We did not have data on inhalers prescribed by non-VA providers. Therefore, we only included patients who were actively using VA inhaler prescriptions prior to and after hospitalization. Findings were similar in sensitivity analyses that included patients who were not receiving inhaler prescriptions for the VA before and/or after hospitalization (Supplementary Table S5). Second, we were not able to ascertain patient's inhaler technique and actual use of these inhalers at home. Although this paper focused on inhaler regimens, other components of COPD care including tobacco cessation, immunizations, and pulmonary rehabilitation are also essential in COPD management. Future interventions to improve delivery of COPD care should also address access to these essential components of COPD care. Third, we excluded patients with concurrent diagnosis of asthma; however, we did not have peripheral blood eosinophil counts to further aid in determining COPD patients who may be appropriately treated with ICS-containing regimens. Fourth, we did not have data regarding in-hospital pulmonary consultation, which may be associated with prescription of guideline-concordant inhaler regimens at discharge. We also did not have data on inhaler dosing. Fifth, our cohort was predominantly male and White which may limit generalizability of the findings. Sixth, the correlations among the exposure variables were among the larger correlations observed in the dataset and the largest present in the table. Rurality and drive time are naturally associated and we anticipated that fragmented care would be associated with the other two exposure variables. Finally, both of our geographic variables have limitations. Our drive time data is limited to the closest VA facility that provides pulmonary specialty care; we did not have data on drive time to services in non-VA settings. Our measure of rurality was broad, and future research is needed to better understand differences in risk within rural populations.

### Conclusion

Living in a rural area, longer drive time to pulmonary specialty care, and fragmented care were associated with higher odds of receiving guideline-discordant inhaler prescriptions after COPD hospitalization. Our findings suggest the need for development of innovative programs to improve delivery of guideline-concordant COPD care, especially in high-risk COPD patients with geographic barriers to care and fragmented care.

## Contributors

Conceived the current analysis and provided supervision: AKB, RAD, KMK, CHW, Designed the analysis: AKB, RAD, KMK, CHW.

Obtained funding: AKB.

Acquired the data: AB.

Performed the data analysis: AKB.

Directly accessed the data: AKB, AB.

Drafted the manuscript: AKB.

Provided critical input and revised the manuscript for important intellectual content and approved the final manuscript: All.

Take responsibility for the integrity of the data and the accuracy of the data analysis: All.

## Data sharing statement

Data sets underlying publications will be shared in electronic format through a de-identified, anonymized dataset. Researchers interested in access to final study data that is not otherwise publicly available would need to prepare a brief (i.e., 3–5 page) research proposal, including the specific sample types and data they wish to obtain. Data sharing will occur under a written agreement that adheres to any applicable informed consent provisions and prohibits the recipient from identifying or re-identifying any individual whose data are included in the dataset. A signed data distribution agreement will be required for each interested researcher, as well as IRB approval, in order to ensure proper procedures of subject protection. project. Data sets will not be shared without first consulting with VA Privacy Officer for approval.

## Declaration of interests

AKB reported grants from the National Institutes of Health. CHS reported grants from the Health Resources and Services Administration, Federal Office of Rural Health Policy, the National Institutes of Health and the CDC; payment or honoraria from Iowa Primary Care Association, Southern Gerontological Society, and the University of Michigan; and leadership or fiduciary role in the National Rural Health Association Board of Trustees. KMK reported personal fees from Nuvaira and Organicell (Data and Safety Monitoring Boards) outside of this work; consulting fees from Allergan/AbbVie and leadership or fiduciary role in the American Thoracic Society Proposal Review Committee, the American Thoracic Society Clinical Problems, and the Assembly Planning Committee.
