# Supplementary material for: Guideline-discordant inhaler regimens after COPD hospitalization: associations with rurality, drive time to care, and fragmented care – a United States cohort study
Source: Lancet Reg Health Am. 2023 Sep 21;26:100597. doi: 10.1016/j.lana.2023.100597 (PMC10520452; doi:10.1016/j.lana.2023.100597)
Supplement: Supplementary Tables [file mmc1.docx]

**ONLINE SUPPLEMENTARY MATERIAL**

Supplemental Data for Article:

**Guideline-Discordant Inhaler Regimens after COPD Hospitalization: Associations with Rurality, Drive Time to Care, and Fragmented Care**

**– a United States Cohort Study**

**Authors:**

Arianne K. Baldomero, MD^1,2,3^, Ken M. Kunisaki, MD^1,2^,

Chris H. Wendt, MD^1,2^, Carrie Henning-Smith, PhD^4^,

Hildi J. Hagedorn, PhD^3^, Ann Bangerter, BS^3^, and R. Adams Dudley, MD^1-3^

^1^Pulmonary, Allergy, Critical Care, and Sleep Medicine, Minneapolis VA Health Care System, Minneapolis, MN, USA

^2^Pulmonary, Allergy, Critical Care, and Sleep Medicine, University of Minnesota, Minneapolis, MN, USA

^3^Center for Care Delivery and Outcomes Research, Minneapolis VA Health Care System, Minneapolis, MN, USA

^4^Division of Health Policy and Management, University of Minnesota, Minneapolis, MN, USA

**Contents of Online Data Supplement**

**Supplementary Table 1.** Summary of Inhaler Regimen Recommendations for Patients with COPD Exacerbations

**Supplementary Table 2.** Logistic Regression Analyses for Guideline-Concordant Inhaler Regimens at 3 Months after COPD Hospitalization (N=33,785)

**Supplementary Table 3.** Logistic Regression Analyses for Individual Inhaler Regimens at 3 Months after COPD Hospitalization (N=33,785)

**Supplementary Figure 1.** Inhaler Regimens at 3 Months and 6 Months after Hospitalization for COPD Exacerbation

**Supplementary Table 4.** Logistic Regression Analyses for Guideline-Discordant Inhaler Regimens 6 Months after COPD Hospitalization (N=31,865)

**Supplementary Table 5.** Logistic Regression Analyses for Guideline-Discordant Inhaler Regimens at 3 Months after COPD Hospitalization (N=58,533)

**Supplementary Table 6.** Logistic Regression Model Combining Rurality, Drive Time, and Fragmented Care for Guideline-Discordant Inhaler Regimens at 3 Months after COPD Hospitalization (N=33,785)

**Full Logistic Regression Models**

**Supplementary Table 7.** Multivariable Logistic Regression: Full Model for Rurality

**Supplementary Table 8.** Multivariable Logistic Regression: Full Model for Drive Time to the Closest Pulmonary Specialty Care

**Supplementary Table 9.** Multivariable Logistic Regression: Full Model for Fragmented Care

**Supplementary Table 10.** Multivariable Logistic Regression: Full Model Including Rurality, Drive Time to Closest Pulmonary Specialty Care, and Fragmented Care

**Multicollinearity Assessments**

**Supplementary Table 11.** Pearson Correlation Coefficients

**Supplementary Table 12.** Tolerance and Variance Inflation

**Supplementary Table 13.** Collinearity Diagnostics

**Secondary Analyses for Sex and Race/Ethnicity**

**Supplementary Table 14.** Logistic Regression Analyses for Guideline-Discordant Inhaler Regimens at 3 Months after COPD Hospitalization by Sex and Race/Ethnicity (N=33,785)

**Interaction Analyses and Stratified Analyses**

**Supplementary Table 15.** Interaction Analyses between Exposure and Explanatory Variables

**Supplementary Table 16.** Logistic Regression Analyses for Guideline-Discordant Inhaler Regimens 3 Months after COPD Hospitalization: Stratified Analyses for Race/Ethnicity and Region by Rurality (N=33,785)

**Supplementary Table 17.** Logistic Regression Analyses for Guideline-Discordant Inhaler Regimens 3 Months after COPD Hospitalization: Stratified Analyses for Race by Fragmented Care (N=33,785)

**STROBE Statement for Cohort Studies**

**Supplementary Table 18.** Strengthening the Reporting of Observational Studies in Epidemiology (STROBE) Statement for Cohort Studies

**Supplementary Table 1. Summary of Inhaler Regimen Recommendations for Patients with COPD Exacerbations**

| **2014 VA/DoD**  **CPG^1^** | We recommend inhaled tiotropium (LAMA) as first-line therapy for patients with confirmed, stable COPD who have respiratory symptoms (e.g., dyspnea, cough) and severe airflow obstruction (i.e., post bronchodilator FEV1 <50%) or a history of COPD exacerbations (strong for).  In patients with confirmed, stable COPD who are on combination therapy with LAMAs (tiotropium) and LABAs and have persistent dyspnea or COPD exacerbations, we suggest adding ICS as a third medication (weak for).  We recommend against offering an inhaled corticosteroid (ICS) in symptomatic patients with confirmed, stable COPD as a first-line monotherapy (strong against). |
| --- | --- |
| **2017 GOLD^2^** | Combination treatment with LAMA+LABA reduces exacerbations compared to monotherapy or combination inhaled corticosteroid/long-acting beta agonist (ICS+LABA) (evidence B).  Triple therapy of LAMA+LABA+ICS improves lung function, symptoms and health status (evidence A) and reduces exacerbations (evidence B) compared to ICS+LABA or LAMA monotherapy.  An ICS combined with a LABA is more effective than the individual components in improving lung function and health status and reducing exacerbations in patients with exacerbations and moderate to severe COPD (evidence A). |
| **2018 GOLD^3^** | LAMAs have a greater effect on exacerbation reduction compared with LABAs (evidence A) and decrease hospitalizations (evidence B).  Combination treatment with a LABA+LAMA reduces exacerbations compared to monotherapy (evidence B) or ICS+LABA (evidence B).  Triple inhaled therapy of ICS+LAMA+LABA improves lung function, symptoms, and health status (evidence A) and reduces exacerbations (evidence B) compared to ICS/LABA or monotherapy. |
| **2019 GOLD^4^**  **2020 GOLD^5^** | LAMAs have a greater effect on exacerbation reduction compared with LABAs (evidence A) and decrease hospitalizations (evidence B).  Combination treatment with a LABA+LAMA reduces exacerbations compared to monotherapy (evidence B).  Triple inhaled therapy of ICS+LAMA+LABA improves lung function, symptoms, and health status, and reduces exacerbations compared to ICS+LABA, LABA+LAMA, or LAMA monotherapy (evidence A). |
| **2020 ATS CPG^6^** | In patients with chronic obstructive pulmonary disease (COPD) who complain of dyspnea or exercise intolerance, we recommend long-acting beta-agonist/long-acting muscarinic antagonist (LABA+LAMA) combination therapy over LABA or LAMA monotherapy (strong recommendation, moderate certainty evidence).  In patients with COPD who complain of dyspnea or exercise intolerance despite dual therapy with LABA/LAMA, we suggest the use of triple therapy with inhaled corticosteroids (ICS)/LABA/LAMA over dual therapy with LABA/LAMA in those patients with a history of one or more exacerbations in the past year requiring antibiotics or oral steroids or hospitalization (conditional recommendation, moderate certainty evidence). |
| **2021 VA/DoD**  **CPG^7^** | We recommend offering inhaled long-acting muscarinic antagonists (LAMA) as first-line therapy in patients with symptomatic COPD (strong for).  We recommend against offering an inhaled corticosteroid (ICS) in patients with symptomatic COPD as a first-line therapy (strong against).  If choosing dual therapy, we recommend *against* offering long-acting beta agonist (LABA) with inhaled corticosteroid (ICS) for patients with COPD (strong against).  In patients with COPD who are on combination therapy with a long-acting anti-muscarinic agent/long-acting beta agonist (LAMA+LABA) and continue to have COPD exacerbations, we suggest adding an inhaled corticosteroid (ICS) as a third medication (weak for). |

*Abbreviations:* VA, Veterans Health Administration; DoD, Department of Defense; CPG, Clinical Practice Guidelines; GOLD, Global Initiative for Chronic Obstructive Lung Disease; ATS, American Thoracic Society

**Supplementary Table 2. Logistic Regression Analyses for Guideline-Concordant Inhaler Regimens at 3 Months after COPD Hospitalization (N=33,785)**

|  | **Guideline-Concordant Inhaler Regimens*^†^**  n=17,387 | |
| --- | --- | --- |
|  | Adjusted Rate, % | Adjusted Odds Ratio |
|  | (95% Confidence Interval) | |
| **Rurality** |  |  |
| Urban (ref) | 51.8 (48.8-54.7) | 1.00 |
| Rural | 47.7 (44.6-50.8) | 0.85 (0.81-0.89) |
| **Drive Time to the Closest Pulmonary Specialty Care** | | |
| ≤30 min (ref) | 53.5 (50.4-56.5) | 1.00 |
| 31-60 min | 51.2 (48.1-54.3) | 0.91 (0.86-0.97) |
| 61-90 min | 45.5 (42.4-48.6) | 0.84 (0.79-0.91) |
| >90 min | 47.0 (44.2-49.8) | 0.73 (0.69-0.77) |
| **Fragmented care**^‡^ |  |  |
| No (ref) | 54.2 (51.2-57.2) | 1.00 |
| Yes | 43.2 (40.2-46.3) | 0.64 (0.61-0.68) |

*Models were adjusted for age, sex, race/ethnicity, Charlson Comorbidity Index (CCI), Area Deprivation Index (ADI), and region.

^†^Guideline-concordant inhaler regimens include: LAMA+LABA or LAMA+LABA+ICS.

^‡^Fragmented care was defined as hospitalization in a non-VA health care facility, but paid for by the VA (VA-purchased care), among patients who receive primary care and prescriptions at the VA.^8^

**Supplementary Table 3. Logistic Regression Analyses for Individual Inhaler Regimens at 3 Months after COPD Hospitalization (N=33,785)***

|  | **Short-Acting**  **Inhalers Only**  n=4,451 | | **ICS+LABA**  n= 7,884 | **LAMA Monotherapy**  n= 3,226 | | | **LAMA+LABA+ICS**  n= 15,574 | |
| --- | --- | --- | --- | --- | --- | --- | --- | --- |
|  | Adjusted Odds Ratio (95% Confidence Interval) | | | | | | | |
| **Rurality** |  | |  |  | | |  | |
| Urban (ref) | 1.00 | | 1.00 | 1.00 | | | 1.00 | |
| Rural | 1.09 (1.04-1.15) | | 1.11 (1.05-1.17) | 1.03 (0.95-1.11) | | | 0.87 (0.83-0.91) | |
| **Drive Time to the Closest Pulmonary Specialty Care** | | | |  |  |  |  |  |
| ≤30 min (ref) | 1.00 | | 1.00 | 1.00 | | | 1.00 | |
| 31-60 min | 1.05 (0.98-1.12) | | 1.06 (0.99-1.14) | 1.04 (0.94-1.14) | | | 0.95 (0.90-1.01) | |
| 61-90 min | 1.02 (0.94-1.11) | | 1.24 (1.14-1.35) | 0.95 (0.84-1.07) | | | 0.88 (0.82-0.94) | |
| >90 min | 1.10 (1.03-1.17) | | 1.37 (1.28-1.46) | 0.95 (0.86-1.05) | | | 0.77 (0.72-0.81) | |
| **Fragmented Care^†^** | |  | |  | | |  | |
| No (ref) | 1.00 | | 1.00 | 1.00 | | | 1.00 | |
| Yes | 1.07 (1.01-1.13) | | 1.39 (1.32-1.47) | 0.99 (0.91-1.07) | | | 0.66 (0.63-0.70) | |

*Abbreviations:* ICS, inhaled corticosteroid; LABA, long-acting beta-agonist; and LAMA, long-acting muscarinic antagonist.

*Models were adjusted for age, sex, race/ethnicity, Charlson Comorbidity Index (CCI), Area Deprivation Index (ADI), and region.

^†^Fragmented care was defined as hospitalization in a non-VA health care facility, but paid for by the VA (VA-purchased care), among patients who receive primary care and prescriptions at the VA.^8^


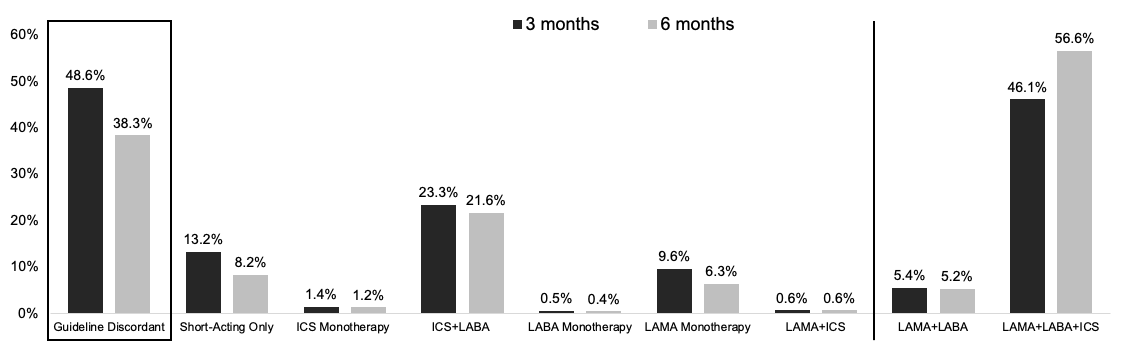


**Supplementary Figure 1. Inhaler Regimens at 3 Months and 6 Months after Hospitalization for COPD Exacerbation.** *Abbreviations:* ICS, inhaled corticosteroid; LABA, long-acting beta-agonist; and LAMA, long-acting muscarinic antagonist.

**Supplementary Table 4. Logistic Regression Analyses for Guideline-Discordant Inhaler Regimens 6 Months after COPD Hospitalization (N=31,865)**

|  | **Guideline-Discordant Inhaler Regimens*^†^**  n=12,188 | |
| --- | --- | --- |
|  | Adjusted Rate, % | Adjusted Odds Ratio |
|  | (95% Confidence Interval) | |
| **Rurality** |  |  |
| Urban (ref) | 39.6 (36.6-42.5) | 1.00 |
| Rural | 43.9 (40.8-47.1) | 1.20 (1.14-1.26) |
| **Drive Time to the Closest Pulmonary Specialty Care** | | |
| ≤30 min (ref) | 38.0 (35.1-41.0) | 1.00 |
| 31-60 min | 40.2 (37.1-43.4) | 1.10 (1.03-1.17) |
| 61-90 min | 42.6 (39.3-46.0) | 1.21 (1.12-1.31) |
| >90 min | 45.3 (42.2-48.6) | 1.35 (1.28-1.44) |
| **Fragmented Care**^‡^ |  |  |
| No (ref) | 36.9 (34.0-39.8) | 1.00 |
| Yes | 48.7 (45.5-51.9) | 1.63 (1.55-1.71) |

*Abbreviations:* ICS, inhaled corticosteroid; LABA, long-acting beta-agonist; and LAMA, long-acting muscarinic antagonist.

*Models were adjusted for age, sex, race/ethnicity, Charlson Comorbidity Index (CCI), Area Deprivation Index (ADI), and region.

^†^Guideline-discordant inhaler regimens include: short-acting inhalers only, ICS monotherapy, ICS+LABA, LABA monotherapy, LABA monotherapy, or LAMA+ICS.

^‡^Fragmented care was defined as hospitalization in a non-VA health care facility, but paid for by the VA (VA-purchased care), among patients who receive primary care and prescriptions at the VA.^8^

**Supplementary Table 5. Logistic Regression Analyses for Guideline-Discordant Inhaler Regimens at 3 Months after COPD Hospitalization (N=58,533)*****

|  | **Guideline-Discordant Inhaler Regimens^†‡^**  n=37,720 | |
| --- | --- | --- |
|  | Adjusted Rate, % | Adjusted Odds Ratio |
|  | (95% Confidence Interval) | |
| **Rurality** |  |  |
| Urban (ref) | 63.8 (61.9-65.7) | 1.00 |
| Rural | 67.4 (65.5-69.3) | 1.18 (1.13-1.22) |
| **Drive Time to the Closest Pulmonary Specialty Care** | | |
| ≤30 min (ref) | 62.3 (60.2-64.2) | 1.00 |
| 31-60 min | 63.8 (61.7-65.8) | 1.07 (1.02-1.12) |
| 61-90 min | 67.2 (65.2-69.3) | 1.25 (1.18-1.32) |
| >90 min | 69.6 (67.7-71.4) | 1.38 (1.33-1.45) |
| **Fragmented care**^§^ |  |  |
| No (ref) | 58.8 (56.7-60.8) | 1.00 |
| Yes | 76.0 (74.4-77.6) | 2.23 (2.14-2.31) |

*Analyses including patients who died before 3 months follow-up and patients who did not receive inhaler prescriptions 3 months before and/or after COPD hospitalization.

^†^Models were adjusted for age, sex, race/ethnicity, Charlson Comorbidity Index (CCI), Area Deprivation Index (ADI), and region.

^‡^Guideline-discordant inhaler regimens include: short-acting inhalers only, ICS monotherapy, ICS+LABA, LABA monotherapy, LAMA monotherapy, or LAMA+ICS.

^§^Fragmented care was defined as hospitalization in a non-VA health care facility, but paid for by the VA (VA-purchased care), among patients who receive primary care and prescriptions at the VA.^8^

**Supplementary Table 6. Logistic Regression Model Combining Rurality, Drive Time, and Fragmented Care for Guideline-Discordant Inhaler Regimens at 3 Months after COPD Hospitalization (N=33,785)**

|  | **Guideline-Discordant Inhaler Regimens*^†^**  n=16,398 | |
| --- | --- | --- |
|  | Adjusted Rate, % | Adjusted Odds Ratio |
|  | (95% Confidence Interval) | |
| **Rurality** |  |  |
| Urban (ref) | 50.8 (47.8-53.8) | 1.00 |
| Rural | 53.0 (49.9-56.1) | 1.10 (1.04-1.15) |
| **Drive Time to the Closest Pulmonary Specialty Care** | | |
| ≤30 min (ref) | 50.5 (47.4-53.6) | 1.00 |
| 31-60 min | 51.0 (47.8-54.1) | 1.02 (0.96-1.08) |
| 61-90 min | 51.4 (48.1-54.7) | 1.04 (0.96-1.12) |
| >90 min | 54.8 (51.7-57.9) | 1.19 (1.12-1.26) |
| **Fragmented care**^‡^ |  |  |
| No (ref) | 46.9 (43.9-49.9) | 1.00 |
| Yes | 56.9 (53.8-59.9) | 1.49 (1.42-1.57) |

*Model was adjusted for rurality, drive time to closest pulmonary specialty care, fragmented care, age, sex, race/ethnicity, Charlson Comorbidity Index (CCI), Area Deprivation Index (ADI), and region.

^†^Guideline-discordant inhaler regimens include: short-acting inhalers only, ICS monotherapy, ICS+LABA, LABA monotherapy, LAMA monotherapy, or LAMA+ICS.

^‡^Fragmented care was defined as hospitalization in a non-VA health care facility, but paid for by the VA (VA-purchased care), among patients who receive primary care and prescriptions at the VA.^8^

**Supplementary Table 7. Multivariable Logistic Regression: Full Model for Rurality**

| **Odds Ratio Estimates and Profile-Likelihood Confidence Intervals** | | | | |
| --- | --- | --- | --- | --- |
| **Effect** | **Unit** | **Estimate** | **95% Confidence Limits** | |
| **Rurality: Rural vs Urban** | 1.0000 | 1.177 | 1.123 | 1.233 |
| **Age** | 1.0000 | 1.005 | 1.003 | 1.008 |
| **Sex: Female vs Male** | 1.0000 | 1.153 | 1.030 | 1.291 |
| **Race: Black vs White** | 1.0000 | 0.851 | 0.797 | 0.909 |
| **Race: Other vs White** | 1.0000 | 1.030 | 0.943 | 1.124 |
| **CCI: 0 vs 3** | 1.0000 | 0.924 | 0.844 | 1.012 |
| **CCI: 1 vs 3** | 1.0000 | 0.811 | 0.775 | 0.849 |
| **Region: Midwest vs West** | 1.0000 | 1.042 | 0.974 | 1.115 |
| **Region: Northeast vs West** | 1.0000 | 0.892 | 0.823 | 0.965 |
| **Region: South vs West** | 1.0000 | 1.121 | 1.053 | 1.193 |
| **ADI** | 1.0000 | 1.004 | 1.003 | 1.005 |

*Abbreviations*: CCI, Charlson Comorbidity Index; ADI, Area Deprivation Index

**Supplementary Table 8. Multivariable Logistic Regression: Full Model for Drive Time to the Closest Pulmonary Specialty Care**

| **Odds Ratio Estimates and Profile-Likelihood Confidence Intervals** | | | | |
| --- | --- | --- | --- | --- |
| **Effect** | **Unit** | **Estimate** | **95% Confidence Limits** | |
| **Drive Time: 30-60 vs ≤30 minutes** | 1.0000 | 1.094 | 1.032 | 1.161 |
| **Drive Time: 61-90 vs ≤30 minutes** | 1.0000 | 1.187 | 1.105 | 1.274 |
| **Drive Time: ≥90 vs ≤30 minutes** | 1.0000 | 1.377 | 1.301 | 1.456 |
| **Age** | 1.0000 | 1.005 | 1.002 | 1.008 |
| **Sex: Female vs Male** | 1.0000 | 1.142 | 1.020 | 1.279 |
| **Race: Black vs White** | 1.0000 | 1.142 | 1.020 | 1.279 |
| **Race: Other vs White** | 1.0000 | 1.142 | 1.020 | 1.279 |
| **CCI: 0 vs 3** | 1.0000 | 0.958 | 0.874 | 1.049 |
| **CCI: 1 vs 3** | 1.0000 | 0.812 | 0.776 | 0.850 |
| **Region: Midwest vs West** | 1.0000 | 0.958 | 0.874 | 1.049 |
| **Region: Northeast vs West** | 1.0000 | 0.812 | 0.776 | 0.850 |
| **Region: South vs West** | 1.0000 | 0.958 | 0.874 | 1.049 |
| **ADI** | 1.0000 | 1.003 | 1.002 | 1.004 |

*Abbreviations*: CCI, Charlson Comorbidity Index; ADI, Area Deprivation Index

**Supplementary Table 9. Multivariable Logistic Regression: Full Model for Fragmented Care**

| **Odds Ratio Estimates and Profile-Likelihood Confidence Intervals** | | | | |
| --- | --- | --- | --- | --- |
| **Effect** | **Unit** | **Estimate** | **95% Confidence Limits** | |
| **Fragmented Care: Yes vs. No** | 1.0000 | 1.555 | 1.481 | 1.632 |
| **Age** | 1.0000 | 1.008 | 1.006 | 1.011 |
| **Sex: Female vs Male** | 1.0000 | 1.129 | 1.007 | 1.264 |
| **Race: Black vs. White** | 1.0000 | 0.851 | 0.798 | 0.907 |
| **Race: Other vs White** | 1.0000 | 0.996 | 0.912 | 1.088 |
| **CCI: 0 vs 3** | 1.0000 | 0.916 | 0.837 | 1.004 |
| **CCI: 1 vs 3** | 1.0000 | 0.809 | 0.773 | 0.847 |
| **Region: Midwest vs West** | 1.0000 | 1.068 | 0.997 | 1.143 |
| **Region: Northeast vs West** | 1.0000 | 0.907 | 0.837 | 0.982 |
| **Region: South vs West** | 1.0000 | 1.145 | 1.076 | 1.219 |
| **ADI** | 1.0000 | 1.004 | 1.003 | 1.005 |

*Abbreviations*: CCI, Charlson Comorbidity Index; ADI, Area Deprivation Index

**Supplementary Table 10. Multivariable Logistic Regression: Full Model Including Rurality, Drive Time to Closest Pulmonary Specialty Care, and Fragmented Care**

| **Odds Ratio Estimates and Profile-Likelihood Confidence Intervals** | | | | |
| --- | --- | --- | --- | --- |
| **Effect** | **Unit** | **Estimate** | **95% Confidence Limits** | |
| **Rurality: Rural vs Urban** | 1.0000 | 1.095 | 1.041 | 1.152 |
| **Drive Time: 30-60 vs ≤30 minutes** | 1.0000 | 1.017 | 0.957 | 1.082 |
| **Drive Time: 61-90 vs ≤30 minutes** | 1.0000 | 1.036 | 0.961 | 1.117 |
| **Drive Time: ≥90 vs ≤30 minutes** | 1.0000 | 1.186 | 1.115 | 1.262 |
| **Fragmented Care: Yes vs. No** | 1.0000 | 1.493 | 1.420 | 1.570 |
| **Age** | 1.0000 | 1.008 | 1.005 | 1.011 |
| **Sex: Female vs. Male** | 1.0000 | 1.137 | 1.015 | 1.273 |
| **Race: Black vs. White** | 1.0000 | 0.898 | 0.841 | 0.960 |
| **Race: Other vs White** | 1.0000 | 0.997 | 0.913 | 1.088 |
| **CCI: 0 vs 3** | 1.0000 | 0.934 | 0.852 | 1.024 |
| **CCI: 1 vs 3** | 1.0000 | 0.809 | 0.773 | 0.847 |
| **Region: Midwest vs West** | 1.0000 | 1.079 | 1.007 | 1.155 |
| **Region: Northeast vs West** | 1.0000 | 0.923 | 0.852 | 1.000 |
| **Region: South vs West** | 1.0000 | 1.155 | 1.084 | 1.230 |
| **ADI** | 1.0000 | 1.003 | 1.002 | 1.004 |

*Abbreviations*: CCI, Charlson Comorbidity Index; ADI, Area Deprivation Inde

**Multicollinearity Analyses**

We assessed multicollinearity among the three exposure variables by examining the correlation matrix (**Supplementary Table 7**) for the variables and by examining variance inflation factors in a multivariable regression model (**Supplementary Table 8-9**). We did not observe high correlation (e.g. correlation coefficients of 0.8 or higher) among the three exposure variables and the variance inflation factors were not large (values were between 1.02 to 1.30, suggesting few issues or problems stemming from multicollinearity in a multivariable model. However, the correlations among the exposure variables were among the larger correlations observed in the dataset and the largest present in the table. Rurality and drive time are naturally associated and we anticipated that fragmented care would be associated with the other two exposure variables.

We performed multivariable logistic regression analyses incorporating a single exposure variable in each model to assess if these exposure variables are each associated with prescription of guideline-discordant inhaler regimens. Analysis of each exposure variable separately could potentially aid in determining geographic risk factors and to identify COPD patients who could be targeted for future interventions/programs to optimize delivery of COPD care. We added this justification in the methods section. We have added the presentation of results from a logistic regression analysis incorporating all three exposure variables to round out this assessment. The results of this analysis, as summarized in **Supplementary Table 12** indicate that each of the exposure variables is associated with the outcome conditional on the inclusion of the other two variables; however, with the large sample sizes the associations observed for drive time and rurality may not be as important as the large association observed for fragmented care.

**Supplementary Table 11. Pearson Correlation Coefficients**

|  | | **Pearson Correlation Coefficients** | | | | | | | | | |
| --- | --- | --- | --- | --- | --- | --- | --- | --- | --- | --- | --- |
|  | **Discordant Inhaler Regimen** | | **Rurality** | **Drive Time** | **Fragmented Care** | **Age** | **Sex** | **Race** | **ADI** | **Region** | **CCI** |
| **Discordant Inhaler Regimen** | 1.00000 | | 0.05373 <.0001 | 0.07626 <.0001 | 0.09843 <.0001 | 0.02303 <.0001 | 0.00801 0.1408 | -0.02493 <.0001 | 0.05521 <.0001 | 0.00238 0.6626 | 0.04793 <.0001 |
| **Rurality** | 0.05373 <.0001 | | 1.00000 | 0.39916 <.0001 | 0.09951 <.0001 | 0.01820 0.0008 | -0.03014 <.0001 | -0.19920 <.0001 | 0.18042 <.0001 | 0.03090 <.0001 | -0.01730 0.0015 |
| **Drive Time** | 0.07626 <.0001 | | 0.39916 <.0001 | 1.00000 | 0.25998 <.0001 | 0.03117 <.0001 | -0.00893 0.1009 | -0.15900 <.0001 | 0.17293 <.0001 | 0.03297 <.0001 | 0.00828 0.1281 |
| **Fragmented Care** | 0.09843 <.0001 | | 0.09951 <.0001 | 0.25998 <.0001 | 1.00000 | -0.11757 <.0001 | 0.02447 <.0001 | 0.09108 <.0001 | 0.03666 <.0001 | 0.04371 <.0001 | -0.03768 <.0001 |
| **Age** | 0.02303 <.0001 | | 0.01820 0.0008 | 0.03117 <.0001 | -0.11757 <.0001 | 1.00000 | -0.11635 <.0001 | -0.05862 <.0001 | -0.08764 <.0001 | 0.01301 0.0169 | 0.12709 <.0001 |
| **Sex** | 0.00801 0.1408 | | -0.03014 <.0001 | -0.00893 0.1009 | 0.02447 <.0001 | -0.11635 <.0001 | 1.00000 | 0.01337 0.0140 | 0.00440 0.4212 | 0.01658 0.0023 | -0.04987 <.0001 |
| **Race** | -0.02493 <.0001 | | -0.19920 <.0001 | -0.15900 <.0001 | -0.06728 <.0001 | -0.05862 <.0001 | 0.01337 0.0140 | 1.00000 | 0.06914 <.0001 | -0.05137 <.0001 | 0.06978 <.0001 |
| **ADI** | 0.05521 <.0001 | | 0.18042 <.0001 | 0.17293 <.0001 | 0.03666 <.0001 | -0.08764 <.0001 | 0.00440 0.4212 | 0.06914 <.0001 | 1.00000 | -0.08608 <.0001 | -0.00813 0.1374 |
| **Region** | 0.00238 0.6626 | | 0.03090 <.0001 | 0.03297 <.0001 | 0.04371 <.0001 | 0.01301 0.0169 | 0.01658 0.0023 | -0.05137 <.0001 | -0.08608 <.0001 | 1.00000 | -0.04731 <.0001 |
| **CCI** | 0.04793 <.0001 | | -0.01730 0.0015 | 0.00828 0.1281 | -0.03768 <.0001 | 0.12709 <.0001 | -0.04987 <.0001 | 0.06978 <.0001 | -0.00813 0.1374 | -0.04731 <.0001 | 1.00000 |

*Abbreviations*: ADI, Area Deprivation Index; CCI, Charlson Comorbidity Index

**Supplementary Table 12. Tolerance and Variance Inflation**

| **Parameter Estimates** | | | | | | | | |
| --- | --- | --- | --- | --- | --- | --- | --- | --- |
| **Variable** | **Label** | **DF** | **Parameter Estimate** | **Standard Error** | **t Value** | **Pr > \|t\|** | **Tolerance** | **Variance Inflation** |
| **Intercept** | Intercept | 1 | 0.19593 | 0.02668 | 7.34 | <.0001 | . | 0 |
| **Rurality** |  | 1 | 0.02252 | 0.00625 | 3.60 | 0.0003 | 0.80402 | 1.24374 |
| **Drive Time** |  | 1 | 0.01323 | 0.00251 | 5.26 | <.0001 | 0.77081 | 1.29733 |
| **Fragmented Care** |  | 1 | 0.09900 | 0.00626 | 15.81 | <.0001 | 0.91336 | 1.09486 |
| **Age** |  | 1 | 0.00186 | 0.00034392 | 5.41 | <.0001 | 0.94436 | 1.05891 |
| **Sex** |  | 1 | 0.03261 | 0.01423 | 2.29 | 0.0219 | 0.98368 | 1.01659 |
| **Race** |  | 1 | -0.01048 | 0.00398 | -2.63 | 0.0085 | 0.92992 | 1.07536 |
| **ADI** |  | 1 | 0.00095043 | 0.00011607 | 8.19 | <.0001 | 0.92597 | 1.07995 |
| **Region** | VISN | 1 | 0.00013969 | 0.00041759 | 0.33 | 0.7380 | 0.98442 | 1.01583 |
| **CCI** | CCI | 1 | 0.01265 | 0.00139 | 9.13 | <.0001 | 0.97394 | 1.02675 |

*Abbreviations*: ADI, Area Deprivation Index; CCI, Charlson Comorbidity Index

**Supplementary Table 13. Collinearity Diagnostics**

|  | **Collinearity Diagnostics** | | | | | | | | | | | |
| --- | --- | --- | --- | --- | --- | --- | --- | --- | --- | --- | --- | --- |
| **Number** | **Eigenvalue** | **Condition Index** |  | **Proportion of Variation** | | | | | | | | |
|  |  |  | **Intercept** | **Rurality** | **Drive Time** | **Fragmented Care** | **Age** | **Sex** | **Race** | **ADI** | **Region** | **CCI** |
| 1 | 6.19967 | 1.00000 | 0.00025640 | 0.00629 | 0.00369 | 0.00639 | 0.00030330 | 0.00120 | 0.00412 | 0.00283 | 0.00460 | 0.00649 |
| 2 | 0.97375 | 2.52325 | 2.012872E-7 | 0.03832 | 0.00175 | 0.00773 | 0.00000109 | 0.73221 | 0.11593 | 0.00000307 | 0.00000559 | 0.00001850 |
| 3 | 0.93048 | 2.58126 | 0.00002425 | 0.05781 | 0.00241 | 0.05264 | 0.00003739 | 0.23164 | 0.44104 | 0.00021634 | 0.00001685 | 0.01017 |
| 4 | 0.64842 | 3.09211 | 0.00009197 | 0.05265 | 0.00007699 | 0.82273 | 0.00017657 | 0.00798 | 0.05799 | 0.00131 | 0.00202 | 0.01550 |
| 5 | 0.49026 | 3.55609 | 0.00038521 | 0.56998 | 0.00109 | 0.00011368 | 0.00056025 | 0.00171 | 0.32626 | 0.00008530 | 0.01867 | 0.11128 |
| 6 | 0.33783 | 4.28387 | 0.00071509 | 0.06881 | 0.00322 | 0.02930 | 0.00073668 | 0.01100 | 0.00193 | 0.00849 | 0.13720 | 0.72964 |
| 7 | 0.20255 | 5.53244 | 0.00077621 | 0.11812 | 0.11827 | 0.01507 | 0.00086690 | 0.00022529 | 0.01495 | 0.12594 | 0.58870 | 0.05508 |
| 8 | 0.13492 | 6.77872 | 0.00149 | 0.06287 | 0.80868 | 0.04657 | 0.00133 | 0.00020574 | 0.03109 | 0.24500 | 0.01323 | 0.00300 |
| 9 | 0.07624 | 9.01737 | 0.02898 | 0.02477 | 0.06054 | 0.00195 | 0.05142 | 0.00000427 | 0.00005052 | 0.56240 | 0.22416 | 0.06701 |
| 10 | 0.00587 | 32.49604 | 0.96727 | 0.00037353 | 0.00027933 | 0.01752 | 0.94457 | 0.01384 | 0.00664 | 0.05373 | 0.01140 | 0.00181 |

*Abbreviations*: ADI, Area Deprivation Index; CCI, Charlson Comorbidity Index

**Supplementary Table 14. Logistic Regression Analyses for Guideline-Discordant Inhaler Regimens at 3 Months after COPD Hospitalization by Sex and Race/Ethnicity (N=33,785)**

|  | **Guideline-Discordant Inhaler Regimens*^†^**  n=16,398 | |
| --- | --- | --- |
|  | Adjusted Rate, % | Adjusted Odds Ratio |
|  | (95% Confidence Interval) | |
| **Sex** |  |  |
| Male | 48.4 (44.8-52.0) | 1.00 |
| Female | 51.7 (47.2-56.2) | 1.14 (1.02-1.28) |
| **Race/Ethnicity** |  |  |
| White | 50.9 (48.0-53.7) | 1.00 |
| American Indian/Alaska Native | 49.3 (43.0-55.6) | 0.94 (0.75-1.18) |
| Asian | 49.0 (36.7-61.4) | 0.93 (0.56-1.51) |
| Black/African American | 45.6 (42.4-48.8) | 0.81 (0.76-0.86) |
| Native Hawaiian/Pacific Islander | 54.0 (46.6-61.1) | 1.13 (0.86-1.49) |
| Unknown/Declined | 51.6 (47.9-55.4) | 1.03 (0.93-1.14) |

*Models were adjusted for age, Charlson Comorbidity Index (CCI), Area Deprivation Index (ADI), and region.

^†^Guideline-discordant inhaler regimens include: short-acting inhalers only, ICS monotherapy, ICS+LABA, LABA monotherapy, LABA monotherapy, or LAMA+ICS.

**Supplementary Table 15. Interaction Analyses between Exposure and Explanatory Variables**

| **Type 3 Analysis of Effects** | | | |
| --- | --- | --- | --- |
| **Effect** | **DF** | **Wald Chi-Square** | **Pr > ChiSq** |
| **Rurality** | 1 | 0.2441 | 0.6213 |
| **Age** | 1 | 16.0293 | <.0001 |
| **Sex** | 1 | 2.4946 | 0.1142 |
| **Race** | 5 | 3.8049 | 0.5778 |
| **ADI** | 1 | 51.5379 | <.0001 |
| **Region** | 4 | 25.9185 | <.0001 |
| **CCI** | 2 | 79.2218 | <.0001 |
| **Rurality*Age** | 1 | 1.2188 | 0.2696 |
| **Rurality*Sex** | 1 | 3.3818 | 0.0659 |
| **Rurality*Race** | 5 | 14.9701 | **0.0105** |
| **Rurality*ADI** | 1 | 0.3563 | 0.5505 |
| **Rurality*Region** | 4 | 38.5842 | **<.0001** |
| **Rurality*CCI** | 2 | 2.5294 | 0.2823 |

| **Type 3 Analysis of Effects** | | | |
| --- | --- | --- | --- |
| **Effect** | **DF** | **Wald Chi-Square** | **Pr > ChiSq** |
| **Drive Time** | 3 | 2.1960 | 0.5327 |
| **Age** | 1 | 9.0417 | 0.0026 |
| **Sex** | 1 | 2.9349 | 0.0867 |
| **Race** | 5 | 9.5359 | 0.0895 |
| **ADI** | 1 | 37.7019 | <.0001 |
| **Region** | 4 | 41.2602 | <.0001 |
| **CCI** | 2 | 66.5667 | <.0001 |
| **Drive Time*Age** | 3 | 5.2946 | 0.1515 |
| **Drive Time*Sex** | 3 | 1.4377 | 0.6967 |
| **Drive Time*Race** | 15 | 20.5462 | 0.1520 |
| **Drive Time*ADI** | 3 | 6.5771 | 0.0867 |
| **Drive Time*Region** | 12 | 20.7032 | 0.0549 |
| **Drive Time*CCI** | 6 | 6.5801 | 0.3614 |

| **Type 3 Analysis of Effects** | | | |
| --- | --- | --- | --- |
| **Effect** | **DF** | **Wald Chi-Square** | **Pr > ChiSq** |
| **Fragmented Care** | 1 | 5.8193 | 0.0159 |
| **Age** | 1 | 19.1273 | <.0001 |
| **Sex** | 1 | 4.0660 | 0.0438 |
| **Race** | 5 | 8.7179 | 0.1209 |
| **ADI** | 1 | 53.5103 | <.0001 |
| **Region** | 4 | 33.7355 | <.0001 |
| **CCI** | 2 | 66.2855 | <.0001 |
| **Fragmented Care*Age** | 1 | 2.6076 | 0.1064 |
| **Fragmented Care*Sex** | 1 | 0.1075 | 0.7430 |
| **Fragmented Care*Race** | 5 | 12.4253 | **0.0294** |
| **Fragmented Care*ADI** | 1 | 0.1224 | 0.7264 |
| **Fragmented Care*Region** | 4 | 4.7116 | 0.3182 |
| **Fragmented Care*CCI** | 2 | 2.6472 | 0.2662 |

**Supplementary Table 16. Logistic Regression Analyses for Guideline-Discordant Inhaler Regimens 3 Months after COPD Hospitalization: Stratified Analyses for Race/Ethnicity and Region by Rurality (N=33,785)**

|  | **Guideline-Discordant Inhaler Regimens*^†^** n=16,398 | | |
| --- | --- | --- | --- |
|  | **Rurality: Rural vs. Urban** | | |
|  | Adjusted Odds Ratio  (95% Confidence Interval) | | |
| **Race/Ethnicity** |  |  |  |
| American Indian/Alaska Native | 1.30 (0.83-2.05) | | |
| Asian | 4.21 (1.00-17.70) | | |
| Black/African American | 1.46 (1.22-1.73) | | |
| Native Hawaiian/Pacific Islander | 0.76 (0.43-1.36) | | |
| White | 1.16 (1.10-1.22) | | |
| Unknown/Declined | 1.20 (0.98-1.47) | | |
| **Region** |  | | |
| Midwest | 1.42 (1.30-1.55) | | |
| Northeast | 1.30 (1.14-1.49) | | |
| South | 1.03 (0.96-1.11) | | |
| West | 1.15 (1.03-1.28) | | |

*Abbreviations:* ICS, inhaled corticosteroid; LABA, long-acting beta-agonist; and LAMA, long-acting muscarinic antagonist.

*Models were adjusted for age, sex, Charlson Comorbidity Index (CCI), and Area Deprivation Index (ADI).

^†^Guideline-discordant inhaler regimens include: short-acting inhalers only, ICS monotherapy, ICS+LABA, LABA monotherapy, LABA monotherapy, or LAMA+ICS.

**Supplementary Table 17.** **Logistic Regression Analyses for Guideline-Discordant Inhaler Regimens 3 Months after COPD Hospitalization: Stratified Analyses for Race by Fragmented Care**^‡^ **(N=33,785)**

|  | **Guideline-Discordant Inhaler Regimens*^†^** n=16,398 | | |
| --- | --- | --- | --- |
|  | **Fragmented Care: Yes vs. No** | | |
|  | Adjusted Odds Ratio  (95% Confidence Interval) | | |
| **Race/Ethnicity** |  |  |  |
| American Indian/Alaska Native | 0.98 (0.62-1.57) | | |
| Asian | 0.91 (0.26-3.24) | | |
| Black/African American | 1.88 (1.62-2.18) | | |
| Native Hawaiian/Pacific Islander | 1.59 (0.89-2.82) | | |
| White | 1.52 (1.44-1.60) | | |
| Unknown/Declined | 1.73 (1.41-2.12) | | |

*Abbreviations:* ICS, inhaled corticosteroid; LABA, long-acting beta-agonist; and LAMA, long-acting muscarinic antagonist.

*Models were adjusted for age, sex, Charlson Comorbidity Index (CCI), Area Deprivation Index (ADI), and region.

^†^Guideline-discordant inhaler regimens include: short-acting inhalers only, ICS monotherapy, ICS+LABA, LABA monotherapy, LABA monotherapy, or LAMA+ICS.

^‡^Fragmented care was defined as hospitalization in a non-VA health care facility, but paid for by the VA (VA-purchased care), among patients who receive primary care and prescriptions at the VA.^8^

**Supplementary Table 18.** Strengthening the Reporting of Observational Studies in Epidemiology (STROBE) Statement for Cohort Studies

|  | Item No | Recommendation | | | | | | | | | | Page  Numbers | | | | | |
| --- | --- | --- | --- | --- | --- | --- | --- | --- | --- | --- | --- | --- | --- | --- | --- | --- | --- |
| **Title and abstract** | 1 | (*a*) Indicate the study’s design with a commonly used term in the title or the abstract | | | | | | | | | | Page 1 | | | | | |
|  |  | (*b*) Provide in the abstract an informative and balanced summary of what was done and what was found | | | | | | | | | | Pages 3-4 | | | | | |
| Introduction | | |  | | | | | | | |  | | | | | |  |
| Background/rationale | 2 | Explain the scientific background and rationale for the investigation being reported | | | | | | | | | | Page 5 | | | | | |
| Objectives | 3 | State specific objectives, including any prespecified hypotheses | | | | | | | | | | Page 5 | | | | | |
| Methods | | | |  | | | | | |  | | | | | |  | |
| Study design | 4 | Present key elements of study design early in the paper | | | | | | | | | | Pages 5-9 | | | | | |
| Setting | 5 | Describe the setting, locations, and relevant dates, including periods of recruitment, exposure, follow-up, and data collection | | | | | | | | | | Pages 5-6 | | | | | |
| Participants | 6 | (*a*) Give the eligibility criteria, and the sources and methods of selection of participants. Describe methods of follow-up | | | | | | | | | | Pages 5-6 | | | | | |
|  |  | (*b*) For matched studies, give matching criteria and number of exposed and unexposed | | | | | | | | | | N/a | | | | | |
| Variables | 7 | Clearly define all outcomes, exposures, predictors, potential confounders, and effect modifiers. Give diagnostic criteria, if applicable | | | | | | | | | | Pages 6-10 | | | | | |
| Data sources/ measurement | 8* | For each variable of interest, give sources of data and details of methods of assessment (measurement). Describe comparability of assessment methods if there is more than one group | | | | | | | | | | Pages 6-9 | | | | | |
| Bias | 9 | Describe any efforts to address potential sources of bias | | | | | | | | | | Pages 9-10 | | | | | |
| Study size | 10 | Explain how the study size was arrived at | | | | | | | | | | N/a | | | | | |
| Quantitative variables | 11 | Explain how quantitative variables were handled in the analyses. If applicable, describe which groupings were chosen and why | | | | | | | | | | Pages 9-10 | | | | | |
| Statistical methods | 12 | (*a*) Describe all statistical methods, including those used to control for confounding | | | | | | | | | | Pages 9-10 | | | | | |
|  |  | (*b*) Describe any methods used to examine subgroups and interactions | | | | | | | | | | Pages 9-10 | | | | | |
|  |  | € Explain how missing data were addressed | | | | | | | | | | N/a | | | | | |
|  |  | (*d*) If applicable, explain how loss to follow-up was addressed | | | | | | | | | | N/a | | | | | |
|  |  | € Describe any sensitivity analyses | | | | | | | | | | Pages 9-10 | | | | | |
| Results | | | | |  | | | |  | | | | | |  | | |
| Participants | 13* | (a) Report numbers of individuals at each stage of study—eg numbers potentially eligible, examined for eligibility, confirmed eligible, included in the study, completing follow-up, and analyzed | | | | | | | | | | Figure 1 | | | | | |
|  |  | (b) Give reasons for non-participation at each stage | | | | | | | | | | Figure 1 | | | | | |
|  |  | (c) Consider use of a flow diagram | | | | | | | | | | Figure 1 | | | | | |
| Descriptive data | 14* | (a) Give characteristics of study participants (eg demographic, clinical, social) and information on exposures and potential confounders | | | | | | | | | | Page 10 and Table 1 | | | | | |
|  |  | (b) Indicate number of participants with missing data for each variable of interest | | | | | | | | | | N/a | | | | | |
|  |  | (c) Summarize follow-up time (eg, average and total amount) | | | | | | | | | | N/a | | | | | |
| Outcome data | 15* | Report numbers of outcome events or summary measures over time | | | | | | | | | | Table 2 and Figure 2 | | | | | |
| Main results | 16 | (*a*) Give unadjusted estimates and, if applicable, confounder-adjusted estimates and their precision (eg, 95% confidence interval). Make clear which confounders were adjusted for and why they were included | | | | | | | | | | Table 2 and Figure 2 | | | | | |
|  |  | (*b*) Report category boundaries when continuous variables were categorized | | | | | | | | | | Page 9 | | | | | |
|  |  | (*c*) If relevant, consider translating estimates of relative risk into absolute risk for a meaningful time period | | | | | | | | | | N/a | | | | | |
| Other analyses | 17 | Report other analyses done—eg analyses of subgroups and interactions, and sensitivity analyses | | | | | | | | | | Pages 9-10 | | | | | |
| Discussion | | | | | |  | |  | | | | | |  | | | |
| Key results | 18 | Summarize key results with reference to study objectives | | | | | | | | | | Pages 12-13 | | | | | |
| Limitations | 19 | Discuss limitations of the study, taking into account sources of potential bias or imprecision. Discuss both direction and magnitude of any potential bias | | | | | | | | | | Pages 16-17 | | | | | |
| Interpretation | 20 | Give a cautious overall interpretation of results considering objectives, limitations, multiplicity of analyses, results from similar studies, and other relevant evidence | | | | | | | | | | Pages 12-17 | | | | | |
| Generalizability | 21 | Discuss the generalizability (external validity) of the study results | | | | | | | | | | Pages 17 | | | | | |
| Other information | | | | | |  |  | | | | | |  | | | | |
| Funding | 22 | Give the source of funding and the role of the funders for the present study and, if applicable, for the original study on which the present article is based | | | | | | | | | | Page 10 | | | | | |

References

1. Department of Veterans Affairs and Department of Defense. VA/DoD Clinical Practice Guideline for the Management of Chronic Obstructive Pulmonary Disease 2014. <https://www.healthquality.va.gov/guidelines/CD/copd/VADoDCOPDCPG.pdf> (accessed 2023 July 28).

2. Global Initiative for Chronic Obstructive Lung Disease. Global Strategy for the Diagnosis, Management, and Prevention of Chronic Obstructive Pulmonary Disease 2017 Report. <https://goldcopd.org/wp-content/uploads/2017/02/wms-GOLD-2017-FINAL.pdf> (accessed 2023 Mar 8).

3. Global Initiative for Chronic Obstructive Lung Disease. Global Strategy for the Diagnosis, Management, and Prevention of Chronic Obstructive Pulmonary Disease 2018 Report. https://goldcopd.org/wp-content/uploads/2017/11/GOLD-2018-v6.0-FINAL-revised-20-Nov_WMS.pdf (accessed 2023 June 28).

4. Global Initiative for Chronic Obstructive Lung Disease. Global Strategy for the Diagnosis, Management, and Prevention of Chronic Obstructive Pulmonary Disease 2019 Report. <https://goldcopd.org/wp-content/uploads/2018/11/GOLD-2019-v1.7-FINAL-14Nov2018-WMS.pdf> (accessed 2023 June 28).

5. Global Initiative for Chronic Obstructive Lung Disease. Global Strategy for the Diagnosis, Management, and Prevention of Chronic Obstructive Pulmonary Disease 2020 Report. <https://goldcopd.org/wp-content/uploads/2019/12/GOLD-2020-FINAL-ver1.2-03Dec19_WMV.pdf> (accessed 2023 June 28).

6. Nici L, Mammen MJ, Charbek E, Alexander PE, Au DH, Boyd CM, Criner GJ, Donaldson GC, Dreher M, Fan VS, Gershon AS. Pharmacologic management of chronic obstructive pulmonary disease. An official American Thoracic Society clinical practice guideline. American Journal of Respiratory and Critical Care Medicine. 2020 May 1;201(9):e56-69. https://doiorg/101164/rccm202003-0625ST.

7. Department of Veterans Affairs and Department of Defense. VA/DoD Clinical Practice Guideline for the Management of Chronic Obstructive Pulmonary Disease 2021. https://www.healthquality.va.gov/guidelines/CD/copd/VADoDCOPDCPGFinal508.pdf (accessed 2023 Mar 8).

8. Rinne ST, Elwy AR, Bastian LA, Wong ES, Wiener RS, Liu CF. Impact of multisystem health care on readmission and follow-up among veterans hospitalized for chronic obstructive pulmonary disease. Medical care. 2017 Jul 1;55(7):S20-5. doi: 10.1097/MLR.0000000000000708.
